# ADHD in a women during (peri)menopause: missed diagnoses and cardiac complaints

**DOI:** 10.1192/j.eurpsy.2024.1666

**Published:** 2024-08-27

**Authors:** J. S. Kooij

**Affiliations:** ^1^Psychiatry, Amsterdam UMC/VUMC, Amsterdam; ^2^Adult ADHD, PsyQ, psycho-medical programs, the Hague, Netherlands

## Abstract

**Introduction:**

(Abstract for the accepted case-based workshop by the NDAL section at EPA)

Women with ADHD are underdiagnosed in mental health care, and although ADHD starts in early childhood, the symptoms and impairment of women with ADHD may only be recognised for the first time during (peri)menopause.

**Objectives:**

The relationship between decreasing levels of estrogen and the interaction with dopamine function in the brain in women with ADHD will be discussed and illustrated by a clinical case vignet of Mary, age 54.

**Methods:**

Mary presents with a history of repeated burnout episodes, mood swings, lifetime difficulty concentrating, planning and organising daily life, restlessness, sleep problems, and cardiac complaints.Mary has been working hard her whole life to overcome all difficulties, but her problem is she can never stop, leading to getting burnout several times. This time she is exhausted and can no longer cope; she is visiting the cardiologist for palpitations, hypertension and a recent myocardial infarction.

**Results:**

After a positive screening for ADHD, based on her lifetime symptoms of inattention, restlessness and impulsivity, as well as mood swings, she is referred to a psychiatrist for assessment of ADHD, mood and sleep problems. The pathophysiology behind this cluster of disorders during (peri)menopause, as well as the treatment options will be discussed based on Mary’s case.

**Conclusions:**

Both ADHD in women, (peri)menopausal mood disorders as well as the heart complaints in women during menopause are underrecognised and undertreated, leading to unneccesary suffering and cardiac death in women. It is time for psychiatry to join forces with cardiology and gynaecology for better recognition, sharing knowledge and multidisciplinary treatment of women with mental disorders such as ADHD during menopausal transition (see www.h3-netwerk.nl).

**Disclosure of Interest:**

None Declared

